# The FinO/ProQ-like protein PA2582 impacts antimicrobial resistance in *Pseudomonas aeruginosa*

**DOI:** 10.3389/fmicb.2024.1422742

**Published:** 2024-06-26

**Authors:** Anastasia Cianciulli Sesso, Armin Resch, Isabella Moll, Udo Bläsi, Elisabeth Sonnleitner

**Affiliations:** ^1^Department of Microbiology, Immunobiology and Genetics, Max Perutz Labs, Center of Molecular Biology, Vienna Biocenter, University of Vienna, Vienna, Austria; ^2^Vienna BioCenter PhD Program, a Doctoral School of the University of Vienna and the Medical University of Vienna, Max Perutz Labs, Vienna Biocenter, University of Vienna, Vienna, Austria

**Keywords:** RNA-binding protein, ProQ, *Pseudomonas aeruginosa*, antibiotic resistance, aminoglycosides, antimicrobial peptides

## Abstract

Bacteria employ small regulatory RNAs (sRNA) and/or RNA binding proteins (RBPs) to respond to environmental cues. In *Enterobacteriaceae*, the FinO-domain containing RBP ProQ associates with numerous sRNAs and mRNAs, impacts sRNA-mediated riboregulation or mRNA stability by binding to 5′- or 3′-untranslated regions as well as to internal stem loop structures. Global RNA-protein interaction studies and sequence comparisons identified a ProQ-like homolog (PA2582/ProQ*_Pae_*) in *Pseudomonas aeruginosa* (*Pae*). To address the function of ProQ*_Pae_*, at first a comparative transcriptome analysis of the *Pae* strains PAO1 and PAO1Δ*proQ* was performed. This study revealed more than 100 differentially abundant transcripts, affecting a variety of cellular functions. Among these transcripts were *pprA* and *pprB*, encoding the PprA/PprB two component system, *psrA*, encoding a transcriptional activator of *pprB*, and *oprI*, encoding the outer membrane protein OprI. RNA co-purification experiments with Strep-tagged *Pae* ProQ protein corroborated an association of ProQ*_Pae_* with these transcripts. In accordance with the up-regulation of the *psrA*, *pprA*, and *pprB* genes in strain PAO1Δ*proQ* a phenotypic analysis revealed an increased susceptibility toward the aminoglycosides tobramycin and gentamicin in biofilms. Conversely, the observed down-regulation of the *oprI* gene in PAO1Δ*proQ* could be reconciled with a decreased susceptibility toward the synthetic cationic antimicrobial peptide GW-Q6. Taken together, these studies revealed that ProQ*_Pae_* is an RBP that impacts antimicrobial resistance in *Pae*.

## Introduction

The opportunistic pathogen *Pseudomonas aeruginosa* (*Pae*) is known to cause a variety of infections that are particularly harmful to immuno-compromised individuals (Beswick et al., 2020). A major obstacle in eradicating these infections is the high intrinsic resistance of *Pae* to a wide range of antibiotics (De Oliveira et al., 2020). In addition, the ability of *Pae* to form biofilms exacerbates antibiotic treatment, owing to restricted penetration and altered physiology (Maurice et al., 2018). Moreover, the metabolic versatility of *Pae* and the production of multiple virulence factors further augment the pathogenicity (Rojo, 2010; Azam and Khan, 2019). These traits are controlled by complex regulatory networks, employing sRNAs and RNA binding proteins (RBPs) (Sonnleitner et al., 2003, 2006; Mulcahy et al., 2008; Sonnleitner and Bläsi, 2014; Zhang et al., 2017; Pusic et al., 2018; Sonnleitner et al., 2020). The best characterized RBP in *Pae* is Hfq that assists sRNA-mediated riboregulation (reviewed in Vogel and Luisi, 2011; Kavita et al., 2018; Pusic et al., 2021), and serves together with the catabolite repression control protein Crc as a translational repressor of many transcripts encoding metabolic genes (Sonnleitner and Bläsi, 2014; Sonnleitner et al., 2018; Dendooven et al., 2023).

The FinO/ProQ family represents a diverse group of proteins that are widespread in α-, β-, and γ-Proteobacteria. It includes both specialized plasmid-encoded regulators, such as FinO, FopA and PcnR as well as chromosome encoded regulators like RocC and ProQ, the latter of which binds to sRNAs and mRNAs (reviewed in Glover et al., 2015; Olejniczak and Storz, 2017; Liao and Smirnov, 2023). In *Escherichia coli* and *Salmonella enterica,* ProQ was shown to associate with ~400 mRNAs and ~ 70 sRNAs (Smirnov et al., 2016; Holmqvist et al., 2018; Melamed et al., 2020). In *E. coli,* the deletion of *proQ* resulted in reduced levels of the proline and glycine betaine transporter ProP, and consequently in a reduced growth rate at high salt concentrations (Chaulk et al., 2011; Kerr et al., 2014). In addition, the *proQ* mutant strain was deficient in biofilm formation (Sheidy and Zielke, 2013), and showed a decrease in virulence (Wang et al., 2023). In *S. enterica*, ProQ modulates the expression of genes involved in motility and virulence (Westermann et al., 2019; Bergman et al., 2024), and impacts persister cell formation (Rizvanovic et al., 2022). Furthermore, ProQ affects the growth rate of *S. enterica* in full broth and minimal media with succinate as the sole C-source under microaerobic conditions (El Mouali et al., 2021), and was shown to be involved in sRNA-mediated riboregulation in this organism (Smirnov et al., 2017).

Structural and functional studies revealed that *E. coli* ProQ consists of the N-terminal FinO domain (NTD) (Smith et al., 2004; Gonzalez et al., 2017), which acts as an electrostatic scaffold for RNA binding (Pandey et al., 2020), and the C-terminal Tudor-like domain (CTD) (Ponting, 1997). Both domains are connected by an unstructured linker that is rich in positively charged amino acid (aa)-residues (Gonzalez et al., 2017). While the NTD is the principal RNA binding site with a preference for highly structured RNAs containing double-stranded regions (e.g., intrinsic terminators), the CTD has a broader RNA binding specificity (Pandey et al., 2020; Stein et al., 2020, 2023).

First hints that *Pae* protein PA2582 represents a ProQ-like FinO domain containing RBP came from sequence comparisons and a gradient profiling by sequencing (Grad-seq) approach performed with exponentially growing *Pae* O1 (Olejniczak and Storz, 2017; Gerovac et al., 2021). PA2582 displays a high sequence homology with the N-terminal FinO-domain of *E. coli* ProQ, including the majority of conserved residues important for RNA binding (Gonzalez et al., 2017; Gerovac et al., 2021). However, it lacks the C-terminal Tudor-like domain but contains a C-terminal extension of 36 aa (Liao and Smirnov, 2023).

In this study, we asked whether PA2582 has a regulatory role in *Pae.* A comparative RNA_Seq_ based transcriptome analysis of a PAO1 PA*2582* deletion mutant and the corresponding wild-type revealed that PA2582 affects more than 100 genes, including the transcripts encoding the transcriptional regulator PsrA, the histidine kinase of the PprA/PprB two-component system (TCS) and the outer membrane protein OprI. Biofilms and liquid cultures of strain PAO1∆*proQ* showed an increased and decreased susceptibility toward the aminoglycosides tobramycin and gentamicin and the cationic peptide GW-Q6, respectively. In accordance with previous findings (de Bentzmann et al., 2012; Tseng et al., 2016), these observations can be reconciled with an increased expression of *pprA*/*pprB* and reduced transcript levels of *oprI*, respectively. RNA co-purification experiments with Strep-tagged PA2582 protein supported an interaction of the protein with these transcripts. Based on these observations and structural comparisons with ProQ homologs of *Enterobacteriaceae*, we propose the name ProQ for PA2582.

## Materials and methods

### Bacterial strains and growth conditions

Strains and plasmids used in this study are listed in Supplementary Table S1. If not indicated otherwise, the cultures were grown aerobically in LB (Lysogeny-broth) medium at 37°C (Miller, 1972). If required, *Pae* and *E. coli* were grown in the presence of 250 μg/mL carbenicillin or 50 μg/mL gentamicin and in the presence of 100 μg/mL ampicillin or 15 μg/mL of gentamicin, respectively. The genes controlled by the P*_tac_*-promoter in plasmid pMMB67HE-derivatives were induced by addition of isopropyl β-D-1-thiogalactopyranoside (IPTG) (1 mM final concentration).

### Construction of strains PAO1Δ*proQ*, PAO1-ProQ_Flag_, PAO1-ProQ_Strep_, and PAO1∆*hfq*∆*proQ*

To construct an in-frame deletion of *Pae proQ* and vectors for chromosomal integration of in-frame fusions of *proQ* to Strep-tag and Flag-tag encoding sequences, respectively, the following procedure was used. Two PCR products flanking the PAO1 *proQ* gene were obtained with chromosomal DNA of PAO1 as template. For the upstream fragment, 754 nucleotide (Δ*proQ*) and 1,285 nucleotide (*proQ*_Strep_/*proQ*_Flag_) long sequences were amplified using primer pairs D181/E181 (Δ*proQ*), D181/H181 (*proQ*_Strep_) and D181/I181 (*proQ*_Flag_), respectively. For the downstream fragment, a 770-nucleotide long sequence was amplified with the primer pair F181/G181. The resulting upstream and downstream fragments were then annealed and used as a template for a second overlapping PCR with oligonucleotides D181 and G181. The resulting PCR amplicons were cleaved with *Pst*I and *Eco*RI and ligated into the corresponding sites of plasmid pEXG2. The generated plasmids pEXG2-Δ*proQ,* pEXG2-*proQ_Flag_* and pEXG2-*proQ_Strep_* were mobilized into PAO1 or PAO1∆*hfq* with the aid of the *E. coli* strain S17-1, and were finally chromosomally integrated through selection for gentamicin resistance. Excision of the vector by a second crossover event was achieved by selection for sucrose insensitive cells, as the pEXG2 vector encodes the *Bacillus subtilis sacB* gene, the product of which -levan sucrose- renders *Pae* sensitive to sucrose (Hmelo et al., 2015). The sequences of all mutagenic oligonucleotides used in this study are provided in Supplementary Table S2. All DNA manipulations were verified by DNA sequencing.

### Construction of plasmids pMMB-*proQ*_Strep_ and pMMB-*proQ*_Flag_

Chromosomal DNA of PAO1-ProQ_Strep_ and PAO1-ProQ_Flag_ were used as templates for PCR amplification together with oligonucleotides P185 and Q185 (Supplementary Table S2). The 663-base pairs (bp) and 657-bp long PCR products, encompassing the *proQ* gene abutted either to the Strep-tag or Flag-tag encoding sequence, were cleaved with *Pst*I and *Eco*RI, and then ligated into the corresponding sites of plasmid pMMB67HE, resulting in plasmids pMMB-*proQ*_Strep_ and pMMB-*proQ*_Flag,_ respectively.

### RNA_Seq_

Total RNA was prepared from two biological replicates of strains PAO1 and PAO1Δ*proQ* grown in LB medium to an OD_600_ of 2.0. Then, 8 mL samples were withdrawn and total RNA was extracted using the hot phenol method (Leoni et al., 1996). The samples were treated with DNase I (TURBO^™^ DNase, Invitrogen), followed by phenol-chloroform-isoamyl alcohol (25:24:1) extraction and ethanol precipitation. Ribosomal RNA was depleted with the NEBNext rRNA Depletion Kit (Bacteria; New England BioLabs). The libraries were constructed using the NEBNext^®^ Ultra^™^ Directional RNA Library Prep Kit for Illumina^®^. Hundred bp single end sequence reads were generated using the Illumina HiSeq 2000 platform at the in-house Next Generation Sequencing Facility (VBCF, Vienna, Austria^1^). Sequencing quality control of the raw reads was assessed using FastQC^2^ software and adaptor sequences were removed with Cutadapt (Martin, 2011). Mapping of the reads against the PAO1 reference genome (NCBI accession number NC_002516.2) was performed with Segemehl (Hoffmann et al., 2009) with default parameters. Reads per gene were counted using BEDTools (Quinlan and Hall, 2010) and the Refseq annotation of *Pae* (NC_002516.2). Differential gene expression analysis was performed with the DESeq2 R package (Love et al., 2014). All genes with a fold-change greater than 2 and a multiple testing adjusted *p*-values below 0.05 were considered to be modulated. The raw sequencing data were deposited in the European Nucleotide Archive (ENA) under accession number PRJEB73792.

### Reverse transcription quantitative polymerase chain reactions

For the real time reverse transcription quantitative polymerase chain reaction (RT-qPCR), PAO1 and PAO1Δ*proQ* were grown in LB medium to an OD_600_ of 2.0. RNA extraction was carried out as described above for the RNA_Seq_ analysis. Then, cDNA was synthesized from 1 μg of DNA-free RNA using random hexamer primers (Promega) and SuperScript III reverse transcriptase (Thermo Fisher) as specified by the manufacturer. The real-time RT-qPCR was performed with 5 × HOT FIREPol EvaGreen^®^ qPCR Mix Plus (no ROX) (Medibena), 25 ng cDNA, and 250 nM of each primer. For all reactions including the DNA standards and the negative control (no template), two biological replicates and three technical replicates were generated. The PCR was performed with specific oligonucleotides for *psrA* (G196/H196), *pprA* (A195/B195), *pprB* (U194/V194), and *cupE1* (S194/T194) (Supplementary Table S2). The transcript levels of the *rpoD* gene obtained with the primer pair Q117/R117 (Supplementary Table S2) were used for normalization of the signals for RT-qPCR as described by Lee et al. (2012). Fold-changes in the *psrA*, *pprA*, *pprB* and *cupE1* mRNA levels were calculated as previously described (Pfaffl, 2001).

### Co-purification of mRNAs with ProQ*_Pae_*

PAO1∆*proQ* harboring either plasmid pMMB67HE (mock control) or pMMB-*proQ*_Strep_ were grown in 1 L LB medium to an OD_600_ of 2.0. The expression of the plasmid encoded *proQ*_Strep_ gene controlled by the P*_tac_* promoter was induced throughout growth with IPTG (1 mM final concentration). The harvested cells were washed with buffer W (100 mM Tris/HCl pH 7.0, 150 mM NaCl, 1 mM EDTA) and further processed by resuspending them in buffer W containing 20 μg/mL lysozyme and 2 mM β-mercaptoethanol. Lysis was accomplished using a single cycle in a cell disruptor (Constant Systems Ltd.) with the pressure set at 1.9 kPa. After lysis, 1 mM phenylmethylsulfonyl fluoride (PMSF) was added. ProQ-Strep was further purified using Strep-Tactin® Sepharose following the protocol provided by the manufacturer (IBA). The RNA bound to ProQ-Strep was subjected to phenol-chloroform extraction and ethanol precipitation. The mock control was treated in the same way as the ProQ-Strep containing samples. For RT-PCR, cDNA was synthesized from 1 μg of DNA-free RNA that was bound to ProQ-Strep as well as from an equivalent volume of the mock control using random hexamer primers (Promega) and SuperScript III reverse transcriptase (Thermo Fisher) as specified by the manufacturer. For RT-PCR, 1 μL of a 1:10 dilution of the RT reaction or the corresponding amount of RNA (5 ng) without addition of reverse transcriptase (negative control) or an equivalent volume of the mock control with and without reverse transcriptase were used with Go-Taq Master Mix (Promega) and 30 cycles of PCR. The PCR was performed with specific oligonucleotides for *psrA* (G196/H196), *pprA* (A195/B195), *pprB* (U194/V194), and *cupE1* (S194/T194) (Supplementary Table S2). The experiment was performed with two biological replicates.

### Northern-blot analysis

The transcript levels of *oprI* were determined by Northern-blotting employing 10 μg of total RNA or 4 μg RNA bound to ProQ-Strep. The RNA samples were denatured for 5 min at 65°C in loading buffer containing 50% formamide, separated on 6% polyacrylamide gels containing 8 M urea, and then transferred to nylon membranes by electroblotting. The RNAs were cross-linked to the membrane by exposure to UV light and then hybridized with an *oprI*-specific ^32^P-labeled oligonucleotide (I84; Supplementary Table S2) at 55°C overnight. A 5S rRNA-specific oligonucleotide (I26; Supplementary Table S2) was used to detect 5S rRNA (loading control). The signals were visualized using a PhosphorImager (Molecular Dynamics).

### Determination of the minimal bactericidal concentration of tobramycin and gentamicin for biofilms grown on glass beads

The MBCs of tobramycin and gentamicin were determined by growing PAO1 and PAO1Δ*proQ* biofilms on glass beads (Konrat et al., 2016). One autoclaved 4 mm glass bead (ROBU^®^ Glasfilter-Geräte GmbH, Hattert, Germany) was placed into each well of a 48 well microtiter plate. The overnight cultures grown in LB medium were diluted to an OD_600_ of 0.05 in LB medium and dispensed into the bead-containing 48 well microplates (1 mL per well). The plate was then placed into a moisture box and incubated at 37°C for 24 h at 120 rpm on an orbital shaker. After 24 h, the liquid culture was removed, and the beads were washed twice with 1x PBS to remove loosely attached bacteria. Fresh medium was added with or without serial dilutions of the respective antibiotic (tobramycin: 3.0-12.0 µg/ml). Gentamicin: 3.0–48.0 μg/mL and incubated for additional 20 h at 37°C at 120 rpm on an orbital shaker. Subsequently, the beads were washed twice with 1x PBS and placed in a 2 mL microcentrifuge tube containing 1 mL of fresh 1x PBS. The samples were vortexed for 30 s, sonicated in an ultrasonic bath at 35 kHz for 20 min at 25°C, and vortexed again for 30 s. The bacterial suspensions were serially diluted in 1x PBS and spotted onto LB agar plates. After 24 h of incubation at 37°C, the biofilm cells were quantified as CFU/bead.

### Antimicrobial activity of the GW-Q6 peptide against PAO1 and PAO1Δ*proQ*

The GW-Q6 peptide (GIKIAKKAITIAKKIAKIYW) was synthesized by ProteoGenix SAS (Schiltigheim, France) with more than 95% purity, and its molecular size was verified by mass spectrometry. The antimicrobial activity was tested as described by Tseng et al. (2016) with the following modifications. PAO1, PAO1Δ*proQ*, PAO1(pMMB67HE), PAO1Δ*proQ*(pMMB67HE), and PAO1Δ*proQ*(pMMB-*proQ*_Flag_) were grown aerobically in LB medium to an OD_600_ of 2.0. The expression of *proQ*_Flag_ in strain PAO1Δ*proQ*(pMMB-*proQ*_Flag_) was induced with IPTG (1 mM final concentration) 30 min before reaching an OD_600_ of 2.0. Cultures of PAO1(pMMB67HE) and PAO1Δ*proQ*(pMMB67HE) were treated with the same concentration of IPTG. Subsequently, approximately 10^4^ cells were either left untreated or treated with a sub-inhibitory concentration (0.1 μM) of the GW-Q6 peptide for 1.5 h at 37°C. Then, serial dilutions were plated on LB agar plates and the corresponding CFU/ml were determined after overnight growth at 37°C. The values of the untreated cells were set to 100% and the percentage of cell survival for the treated cells were calculated accordingly.

### Determination of OprI levels

PAO1, PAO1Δ*proQ*, PA14, and PA14Δ*oprI* strains were grown in 25 mL LB medium to an OD_600_ of 2.0. Then, the cells were harvested by centrifugation at 5,000 × g for 10 min at 4°C, resuspended in 5 mL 10 mM HEPES buffer (pH 7.8) and lysed by sonication. Cell debris were removed by centrifugation at 20,000 × g for 30 min. The cleared lysates were collected and centrifuged at 200,000 × g for 1 h at 4°C. The supernatants, which represents the cytoplasmic fraction, were discarded. The pellets, which contained the total membrane protein fraction, were dissolved in 500 μL of 10 mM HEPES buffer (pH 7.8) supplemented with N-lauryl sarcosine (final concentration 0.7%). The samples were incubated for 30 min at room temperature, and subsequently centrifuged at 200,000 × g for 2 h at 4°C. The resulting supernatants contained the inner membrane (IM) proteins, whereas the pellet fraction comprised the outer membrane (OM) proteins. The OM protein fractions were resuspended in 500 μL of 10 mM HEPES buffer (pH 7.8) containing 2% Triton-X100. The concentration of the OM proteins was assessed with the Pierce^™^ BCA Protein Assay Kit (Thermo Fisher). 15 μg of OM proteins were boiled in Laemmli-buffer (125 mM Tris/HCl pH 6.8, 3% (w/v) SDS, 10% (v/v) glycerol, 5% (v/v) β-mercaptoethanol and 0.0025% (w/v) Bromophenol blue), and separated on a 15.3% Tricine-SDS-polyacrylamide gel containing 4 M urea as described by Schägger (2006). The proteins were visualized by Coomassie Brilliant Blue R 250 staining and the protein levels were quantified with ImageQuantTL software.

### Statistical analysis

Unless indicated otherwise, all experiments were performed in duplicate with two biological replicates. All statistical analyses were performed using GraphPad Prism 8. Except for the antimicrobial activity assay of the synthetic peptide GW-Q6, the statistical analyses were performed with a two-tailed distributed Student’s *t*-test, ns (non-significant); ^∗^*p* < 0.05, ^∗∗^*p* < 0.01, and ^∗∗∗^*p* ≤ 0.001. Due to the multiple comparisons for the antimicrobial activity assay of the synthetic peptide GW-Q6, the results were statistically analyzed by one-way ANOVA with the Tukey’s *post hoc* test. ns (non-significant); ^∗^*p* < 0.05, ^∗∗^*p* < 0.01, and ^∗∗∗^*p* < 0.001 were considered as statistically significant.

## Results

### PA2582 is a ProQ-like protein

**Figure 1:**
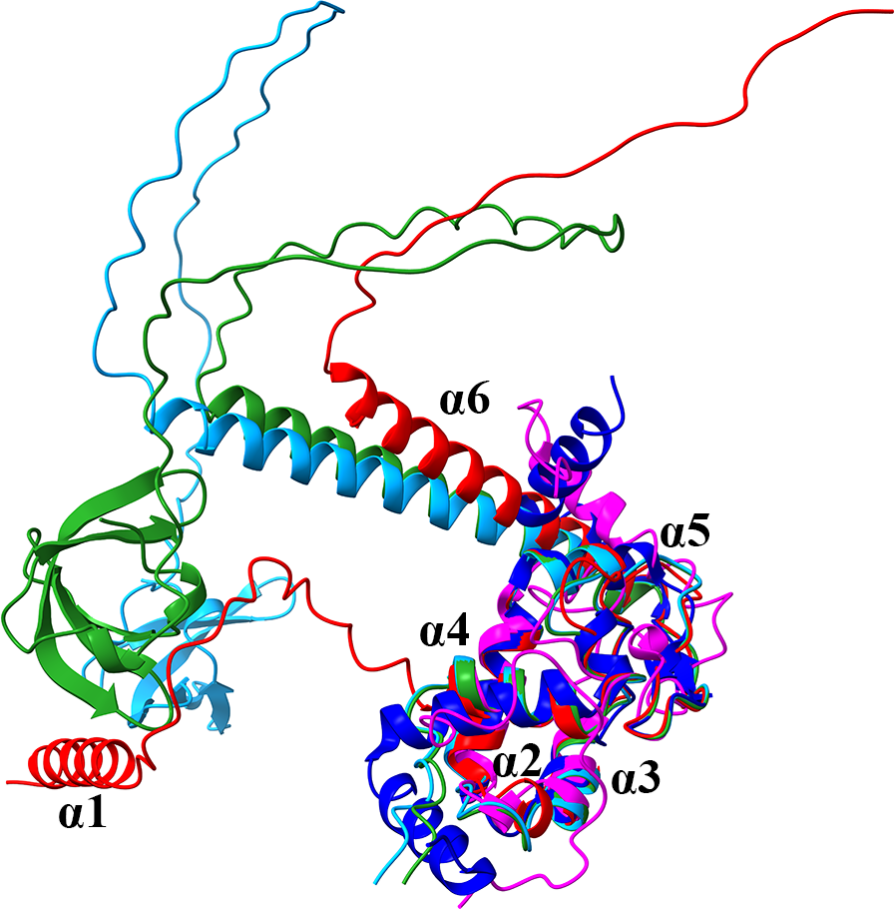
Superposition of the ribbon diagrams of the predicted AlphaFold structures of ProQ*_Pae_* (Q9I0Q4; red), the ProQ homologs of *E. coli* (P45577; light blue) and *S. enterica* (A0A3Y9V7K5; green), the N-terminal ProQ NMR structure of *E. coli* (PDB ID: 5NB9; magenta) and the crystal structure of ProQ*_Nme_* from *N. meningitidis* (PDB ID: 3 MW6; dark blue).

**Figure 2:**
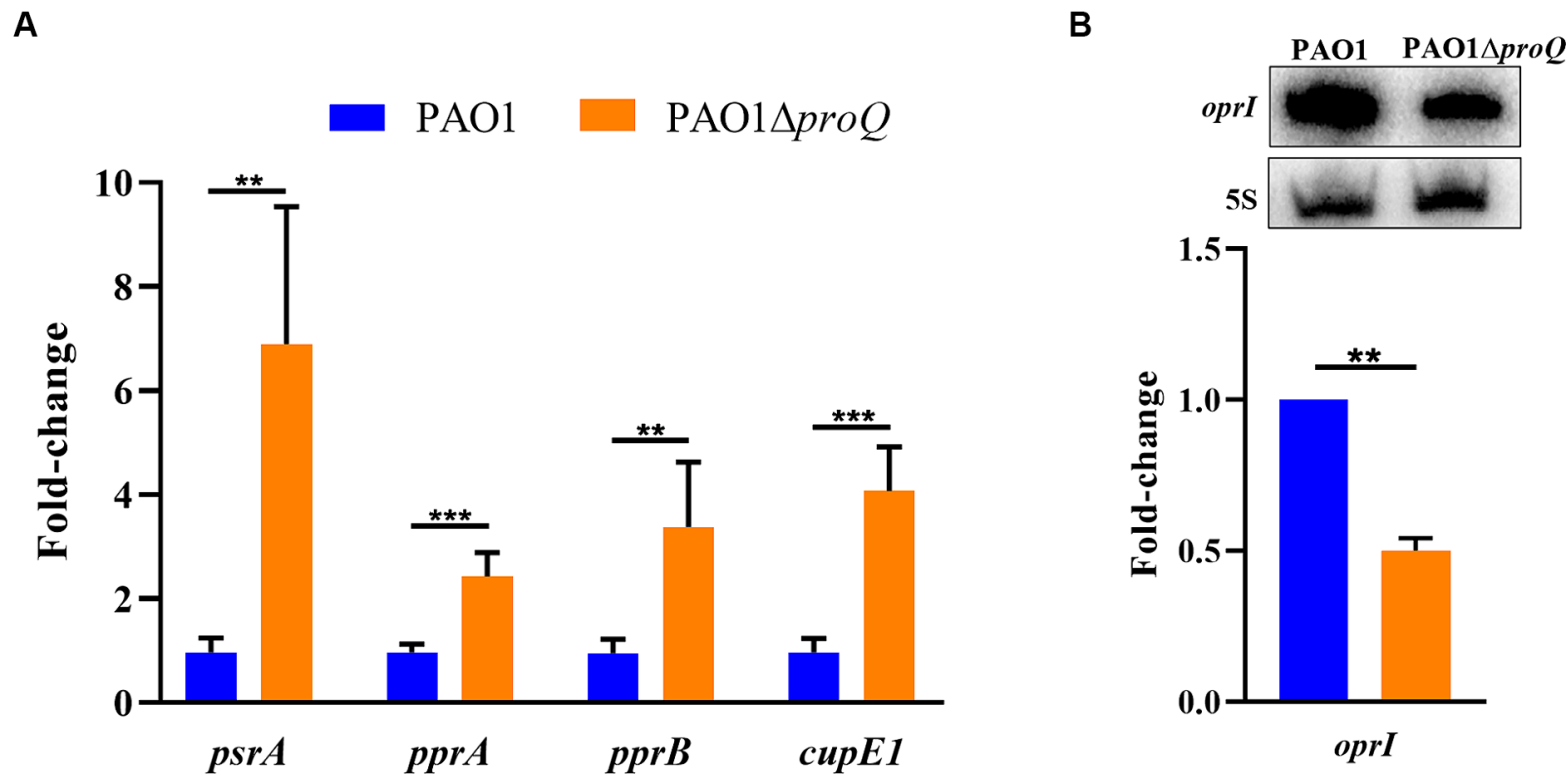
ProQ*_Pae_* affects the transcript levels of *psrA*, *pprA*, *pprB*, and *cupE1*. **(A)** Total RNA was purified from cultures of PAO1 and PAO1Δ*proQ* after growth in LB medium to an OD_600_ of 2.0. The RT-qPCR was carried out with three technical replicates derived from two biological replicates and is given as fold-change relative to PAO1. Error bars represent standard deviations from two biological replicates, each performed in triplicate. Significance was evaluated using a two-tailed Student’s *t*-test and indicated as follows: ^∗∗^*p* ≤ 0.01 and ^∗∗∗^*p* ≤ 0.001. **(B)** The Northern-blot signals for *oprI* mRNA were normalized to the signals of 5S rRNA (loading control). A representative picture of a Northern-blot is shown on top. Bottom, graphical representation of the data. The error bar represents the standard deviation from two biological replicates.

To gain initial information whether PA2582 is a ProQ-like protein, the structures predicted by AlphaFold of PA2582 and the ProQ homologs of *E. coli* (ProQ*_Eco_*) and *S. enterica* (ProQ*_Sen_*), the NMR structure of the N-terminal FinO-domain of ProQ*_Eco_* and the crystal structure of ProQ (NMB1681) of *Neisseria meningitidis* (ProQ*_Nme_*) (Chaulk et al., 2010; Gonzalez et al., 2017; Jumper et al., 2021; Varadi et al., 2024) were superimposed (Figure 2). As anticipated from previous sequence comparisons (Gerovac et al., 2021; Liao and Smirnov, 2023), the moiety of PA2582 comprising amino acids 40–122 (α-helix 2 to 5; Supplementary Figure S1A) has a high structural similarity with the FinO-domain of all proteins (Figure 1). The α-helix 6 consists of one long helix and ends in a long unstructured stretch, comparable to ProQ of *E. coli* and *S. enterica*, but lacking the Tudor-like domain. On the other hand, the 31 aa extension at the N-terminus forms an α-helix connected to the FinO-domain with a seemingly unstructured linker. A N-terminal α-helix is also present in ProQ*_Nme_*, which however is devoid of the linker (Figure 1). As described for other ProQ homologs (Olejniczak and Storz, 2017), the electrostatic surface potential of PA2582 shows that parts of α-helix 1 and the inner side of the U-shape protein region formed by the linker between α-helix 1 and the FinO domain, the FinO domain itself and the α-helix 6 are positively charged, which could suggest an involvement in RNA binding (Supplementary Figure S1B). In addition, the conserved residues Y70 and R80 (*E. coli* numbering) that form the main RNA-binding site on this conserved concave face of ProQ*_Eco_* (Stein et al., 2023) are present in PA2582 (corresponding to Y101 and R111 in PA2582). The third essential residue for RNA binding, R58 (*E. coli* numbering) most likely corresponds to K89 in ProQ*_Pae_*, thus retaining the positive charge (Stein et al., 2023; Supplementary Figure S1A). As PA2582 shows significant structural similarities to known ProQ homologs, we henceforward term this protein ProQ*_Pae_*.

### ProQ*_Pae_* is not involved in osmoregulation, oxidative stress response, motility and biofilm formation

In *E. coli* and *S. enterica*, ProQ is known to play a role in a wide range of biological processes including osmotic stress responses, motility and biofilm formation (Sheidy and Zielke, 2013; Kerr et al., 2014). ProQ of *N. meningitidis*, which lacks the Tudor-like domain like ProQ*_Pae_*, was shown to interact with RNAs and to protect transcripts from degradation. In addition, ProQ*_Nme_* is important to survive oxidative stress caused by H_2_O_2_ exposure (Bauriedl et al., 2020). To examine whether ProQ*_Pae_* is as well involved in these processes, we first tested whether ProQ*_Pae_* is important to cope with osmotic stress by growing PAO1 and PAO1Δ*proQ* on microtiter plates in LB medium containing either 0.17 M NaCl (no salt stress), 0.8 M (high salt) or 1.0 M NaCl (osmotic stress). As shown in Supplementary Figure S2A, we observed no growth difference between the two strains in response to high salt concentrations. Similarly, as observed for the growth in LB medium in microtiter plates without salt stress (Supplementary Figure S2A), we noted no growth difference between the two strains grown aerobically in Erlenmeyer flasks (not shown). Second, to test whether ProQ*_Pae_* plays a role in oxidative stress, the growth inhibition zones for PAO1 and PAO1∆*proQ* were determined in the presence of filter disks containing 10 μL of 30% H_2_O_2_. As shown in Supplementary Figure S2B, no difference in the diameter of the inhibition zones was observed. Third, PAO1 and PAO1Δ*proQ* showed the same swimming, swarming and twitching motility (Supplementary Figure S2C), and displayed no significant difference in the production of static biofilm after 24 h (Supplementary Figure S2D). In *N. meningitidis* deletion of *hfq* but not *proQ* reduced growth. However, inactivation of *proQ* in the *hfq* mutant led to further retardation of growth, suggesting that ProQ in *N. meningitis* is complementary but not fully exchangeable with Hfq (Bauriedl et al., 2020). To test whether this growth behavior holds for *Pae,* the growth of PAO1, PAO1Δ*hfq*, PAO1Δ*proQ*, and PAO1Δ*hfq*Δ*proQ* was monitored on microtiter plates in LB medium. Again, there was no significant difference in growth between PAO1Δ*hfq* and PAO1Δ*hfq*Δ*proQ*, and only the Hfq deficiency resulted in a growth defect (Supplementary Figure S2E).

### ProQ*_Pae_* affects the abundance of transcripts encoding diverse proteins

To obtain information on the potential role of ProQ*_Pae_* as a gene expression regulator, a comparative RNA_Seq_ based transcriptome analysis was performed with strains PAO1 and PAO1Δ*proQ*. Prior to this study, we tested whether ProQ*_Pae_* is constitutively produced throughout growth. The protein levels were determined in strain PAO1-ProQ_Flag_, carrying the chromosomally encoded ProQ*_Pae_* protein fused in frame to a C-terminal Flag-tag. The strain PAO1-ProQ_Flag_ was grown aerobically in LB medium and protein samples were collected at an OD_600_ of 1.0 (exponential phase), 2.0 (early stationary phase) and 3.0 (stationary phase), respectively. As shown in Supplementary Figure S3, the ProQ_Flag_ levels did not significantly change throughout growth.

The samples for the RNA_Seq_ analysis were withdrawn at an OD_600_ of 2.0 after aerobic growth of both strains in LB medium. This cell density was chosen with the rationale that regulatory RBPs are known to be employed to cope with stress, which increases by entering stationary phase. For differential gene expression analysis and interpretation, only annotated genes deposited in the *Pseudomonas* genome database (Winsor et al., 2016) were considered for comparison, while the Benjamin-Hochberg adjusted *p*-values (padj) (Benjamini and Hochberg, 1995) of 0.05 was set as a threshold for significance. Only transcripts with a fold-change equal to or greater than ±2 were considered differentially expressed. When compared with PAO1, a total of 161 transcripts were differentially abundant in PAO1∆*proQ*, with 63 and 97 genes being up- and down-regulated, respectively (Supplementary Table S3).

The functional classes representing the majority of down-regulated genes are related to translational and post-translational modification, whereas chaperone proteins were found to be mostly up-regulated in the absence of ProQ*_Pae_*. Furthermore, ProQ*_Pae_* apparently impacts gene functions encoding transcriptional regulators as well as functions involved in fatty acid-, phospholipid-, energy-, central intermediary-, carbon- and amino acid- metabolism. Interestingly, functions involved in transport of small molecules, protein secretion/export apparatus, membrane proteins and cell wall/lipopolysaccharide/capsule synthesis were also found to be differentially expressed (Supplementary Figure S4). On the other hand, none of the annotated *Pae* sRNAs (Winsor et al., 2016) were affected by the absence of ProQ*_Pae_* (Supplementary Table S3).

### ProQ*_Pae_* alters the abundance of genes linked to antimicrobial resistance

As *Pae* is notorious for its high resistance against clinically used antibiotics, we focused in the follow-up studies on differentially regulated genes implicated in antibiotic susceptibility and membrane permeability. The most up-regulated gene in PAO1Δ*proQ* when compared to PAO1 was *psrA* (fold-change of 6.4) (Table 1; Supplementary Table S3). The expression of *psrA* is increased in the presence of cationic antimicrobial peptides (AMPs) (Gooderham et al., 2008). Moreover, a *psrA* mutant showed enhanced susceptibility to the AMPs polymyxin B and indolicidin, which correlated with an OM that was more easily permeabilized by these AMPs in the *psrA* mutant when compared with the wild-type strain. In addition, PsrA functions as a global regulator influencing biofilm formation, type III secretion, adhesion, and swarming motility. It acts as an autogenous repressor and activator of *rpoS* expression (Kojic et al., 2005; Gooderham et al., 2008). Furthermore, a previous microarray analysis revealed PsrA as a positive regulator of *pprB* expression encoding the response regulator of the PprA/PprB TCS (Gooderham et al., 2008). Overexpression of *pprB* resulted in increased susceptibility to aminoglycosides and hyper-biofilm formation that also led to increased susceptibility to tobramycin (Wang et al., 2003; de Bentzmann et al., 2012). When compared with PAO1, the transcript abundance of *pprB* was 1.7-fold increased in PAO1Δ*proQ*, and thus below the set threshold level. However, the corresponding two-component sensor kinase encoding gene *pprA* was 2.59-fold increased (Table 1; Supplementary Table S3). In addition, the transcript PA*4294*, forming an operon together with *pprA*, as well as the *bapA* and *cupE1* genes that are known to be controlled by PprA/PprB, were up-regulated in the absence of ProQ (Table 1; Supplementary Table S3). Furthermore, the transcript encoding the envelope stress response regulator PA4596 (EsrC) was more than 2-fold up-regulated in the absence of ProQ*_Pae_* (Table 1; Supplementary Table S3). The expression of *esrC* is induced under envelope stress conditions. Together with the transcriptional regulator NfxB, it functions as a second repressor of the MexCD-OprJ multi drug resistance operon (Purssell et al., 2015; Lorusso et al., 2022). The MexCD-OprJ efflux system is mainly associated with the resistance to fluoroquinolones but can also extrude other antimicrobial agents (Lorusso et al., 2022). In addition, the transcript PA*3584* (*glpD*), encoding the glycerol-3-phosphate-dehydrogenase, was 4.66-fold upregulated in the absence of ProQ*_Pae_*. A deletion of *glpD* resulted in increased persister cell formation after exposure to ofloxacin (Shuman et al., 2018).

**Table 1:** Selection of genes related to antimicrobial resistance that were differently expressed in PAO1Δ*proQ* versus PAO1.

PA-number	Gene name	Description	Fold-change	padj	Predicted intrinsic terminators (nucleotides after the stop codon)
PA1874	*bapA*	BapA adhesin	2.36	1.26E-06	
PA2853	*oprI*	Outer membrane lipoprotein OprI precursor	−2.70	8.32E-05	**AAAA**CCGGUCCCUCGGGGCCGGUUUUUUU (((((((((((....)))))))))))... (+20 to +48)
PA3006	*psrA*	Transcriptional regulator PsrA	6.41	3.73E-17	**A**G**A**CGGCGCCCCAGGGCGCCGUUUU
PA3584	*glpD*	Glycerol-3-phosphate dehydrogenase	4.66	1.20E-08	
PA4293	*pprA*	Two-component sensor PprA	2.59	3.36E-05	**AAAAAAA**CGCCUGCGGACAAGCAGGCGUUUUUU
PA4294	PA*4294*	Putative pilus assembly protein	2.05	2.12E-03	
PA4596	*esrC*	Envelope stress response regulator	2.26	4.57E-02	
PA4648	*cupE1*	Pilin subunit CupE1	2.72	1.84E-04	
PA4773	*speD2*	Putative S-adenosylmethionine decarboxylase proenzyme	−2.71	4.76E-05	
PA4774	*speE2*	Putative spermidine synthase	−2.03	4.87E-03	

The *oprI* gene, encoding the major OM protein I (OprI) was 2.7-fold down-regulated in the absence of ProQ*_Pae_* (Table 1; Supplementary Table S3). OprI recruits and affects the susceptibility to α-helical AMPs, like GW-Q6 (Lin et al., 2010; Chang et al., 2015; Tseng et al., 2016). In addition, two transcripts involved in the synthesis of the polyamine spermidine, PA*4773* (*speD2*) and PA*4774* (*speE2*), showed reduced abundance in PAO1Δ*proQ* when compared with the wild type (Table 1; Supplementary Table S3). Spermidine contributes to polymyxin susceptibility by interacting with divalent cation-binding sites of lipopolysaccharides (LPS), which renders them inaccessible for polymyxin binding (Johnson et al., 2012). Moreover, inactivation of the gene *speE2* alters the outer membrane permeability barrier to polymyxin B, CP10A, and gentamicin (Johnson et al., 2012). Taken together, ProQ*_Pae_* modulates the expression of genes involved in intrinsic and adaptive antibiotic resistance, primarily influencing functions related to membrane composition and permeability.

### ProQ*_Pae_* interacts with transcripts encoding functions linked to antimicrobial resistance

To verify the RNA_Seq_ data, we next confirmed in strain PAO1Δ*proQ* the increased transcript levels of *psrA*, *pprA, pprB*, and *cupE1* by RT-qPCR (Figure 2A) and the decreased transcript level of *oprI* by Northern-blotting (Figure 2B).

To gain further information on the potential interaction of ProQ*_Pae_* with these transcripts, we first inspected the data of the recently published Grad-seq analysis of *Pae* (Gerovac et al., 2021). This study investigated the interactions between RNA molecules and protein complexes at a global level. Native cellular lysates including RNA-protein complexes were partitioned on a glycerol gradient, fractionized and analyzed by sequencing and mass spectrometry (Gerovac et al., 2021). The majority of the ProQ*_Pae_* protein was found in fractions 3–11, which included the *oprI* (fractions 3 and 4), *psrA* (fractions 3–8), *pprA* (fractions 4 and 5), *pprB* (fraction 4) and *cupE1* (fractions 4 and 7) transcripts (Supplementary Table S4; Gerovac et al., 2021). This re-assessment indicated that ProQ*_Pae_* may indeed associate with these transcripts. Moreover, in *E. coli* and *S. enterica* several RNA ligands of ProQ have A-rich motifs at the 5′-side of intrinsic terminator hairpins (Stein et al., 2020). Therefore, we asked whether the identified putative ProQ targets (Table 1) possess such sequences. As shown in Table 1, at least for *oprI*, *psrA* and *pprA* such intrinsic terminator sequences are present.

**Figure 3:**
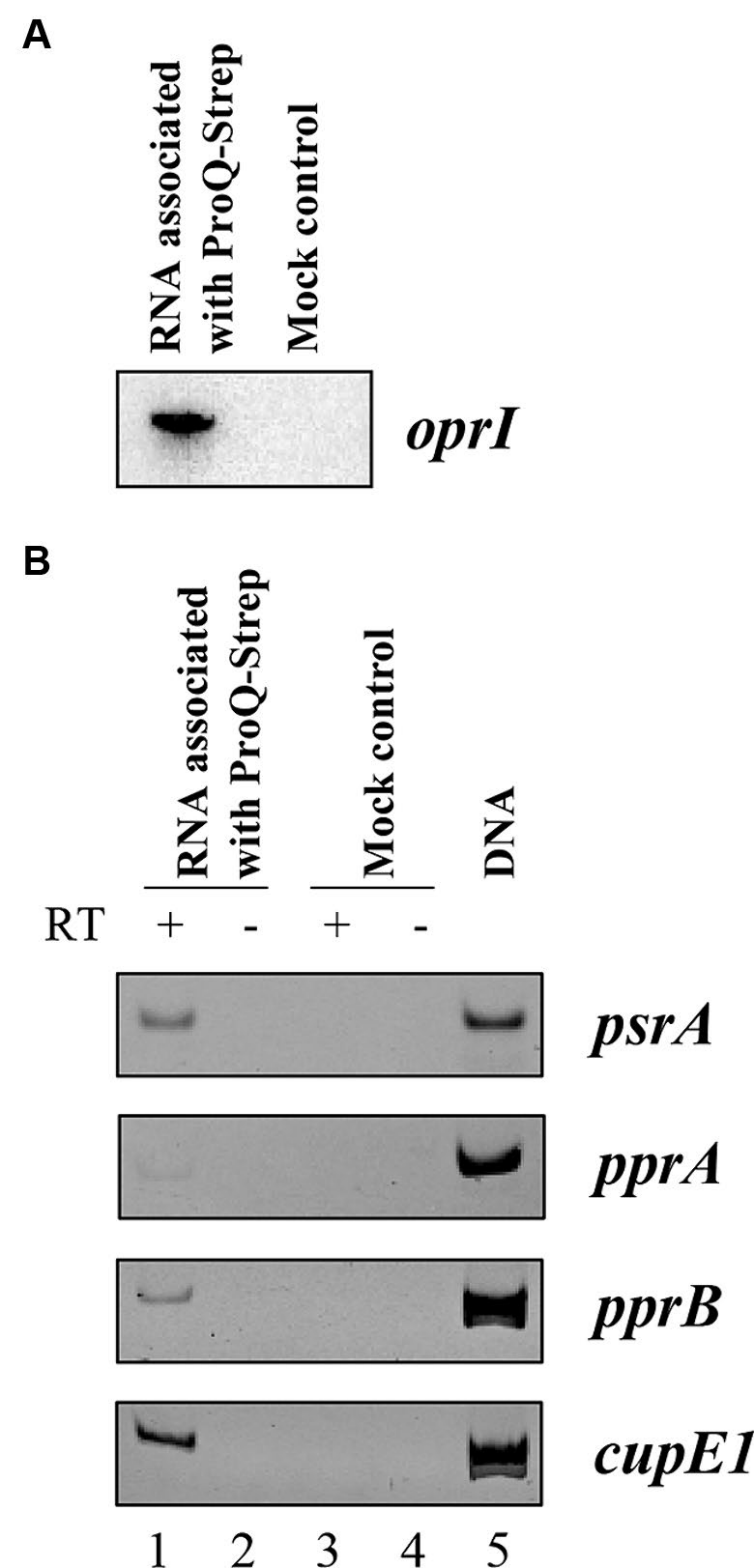
Co-purification of *oprI*, *psrA*, *pprA*, *pprB*, and *cupE1* mRNAs with Strep-tagged ProQ*_Pae_*. Aliquots of the RNA samples associated with ProQ*_Pae_*-Strep were used for Northern-blotting to detect *oprI* mRNA **(A)**, and for RT-PCR to detect *psrA*, *pprA*, *pprB*, and *cupE1* mRNAs **(B)**. PAO1∆*proQ*(pMMB-*proQ*_Strep_) and PAO1∆*proQ*(pMMB67HE) (mock control) were grown in LB medium to an OD_600_ of 2.0. The RNA was isolated by phenol-chloroform extraction after ProQ-Strep protein purification by affinity chromatography using the Strep-Tactin^®^ resin. The corresponding eluates of the mock control were obtained under the same conditions. PCR reactions with the RNA as template without reverse transcriptase reaction were used as negative control (lanes 2 and 4). Genomic DNA served as template for the positive control (lane 5).

To show that ProQ*_Pae_* associates with the *oprI*, *psrA*, and *pprA* mRNAs, we next analyzed RNAs that were bound to Strep-tagged ProQ*_Pae_* protein. PAO1Δ*proQ* harboring plasmid pMMB-*proQ*_Strep_ was grown in LB medium to an OD_600_ of 2.0 and the RNAs bound to ProQ*_Pae_*-Strep were purified as described in Materials and Methods. Unspecific binding to the affinity matrix was controlled by a mock purification using strain PAO1Δ*proQ* harboring the parental plasmid pMMB67HE. After electrophoretic separation and ethidium bromide staining RNA was only visible in the ProQ-Strep derived sample but not in the mock control (Supplementary Figure S5). The presence of *oprI* was confirmed by Northern-blotting (Figure 3A) and that of *psrA* by RT-PCR (Figure 3B). For *pprA*, we only observed a weak signal by RT-PCR, questioning whether *pprA* is a direct target of ProQ*_Pae_.* In addition, the presence of *pprB* and *cupE1* mRNAs was confirmed in the ProQ-Strep derived sample. None of these five mRNAs were detected by RT-PCR in the samples of the mock control. Taken together, these studies strongly indicated that ProQ*_Pae_* can act as an RBP.

### ProQ*_Pae_* affects tobramycin and gentamicin susceptibility in biofilms

According to the studies presented above, ProQ*_Pae_* binds to and affects the abundance of the transcript *psrA* (Table 1; Figures 2A, 3B; Table 1; Supplementary Table S3). The lack of PsrA resulted in an increased membrane permeability and susceptibility to AMPs (Gooderham et al., 2008). To test whether ProQ*_Pae_* affects the susceptibility to AMPs, we determined the minimal inhibitory concentration of colistin in PAO1 and PAO1Δ*proQ* grown in LB medium. However, PAO1 and PAO1Δ*proQ* showed no difference in their susceptibility toward colistin (not shown).

**Figure 4:**
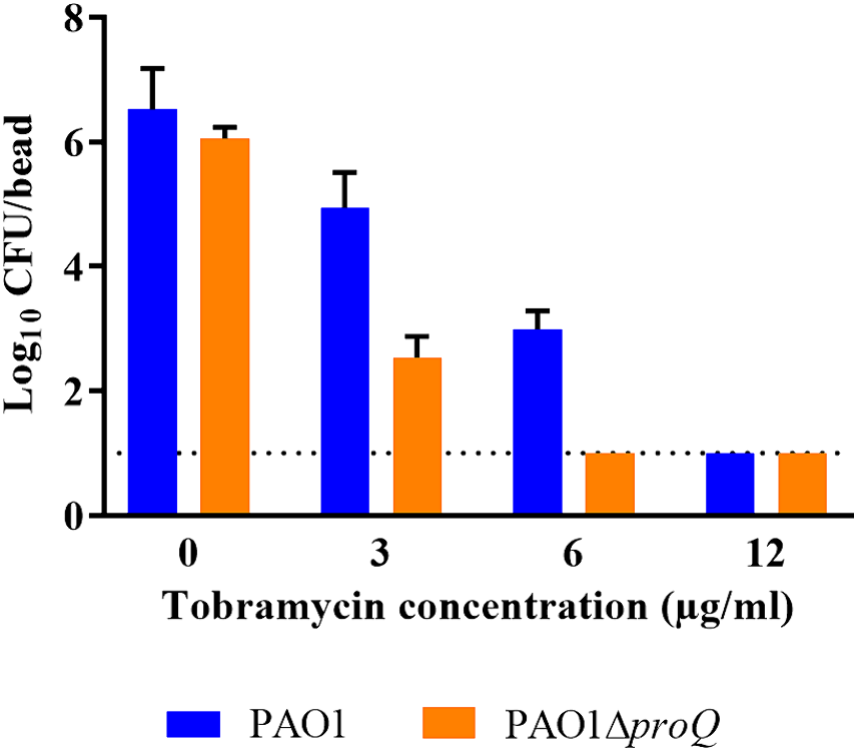
Determination of the minimal bactericidal concentration (MBC) of tobramycin in biofilms. Biofilms of PAO1 and PAO1Δ*proQ* were grown at 37°C on 4 mm glass beads submerged in LB medium as described in Materials and Methods. Biofilm formation is displayed as the logarithm of the CFU per glass bead (Log_10_ CFU/bead). Whenever the CFU count was zero, the value “1” (dashed line) was assigned. The error bars represent standard deviations from two biological replicates.

As mentioned above, PsrA was identified as a positive regulator of *pprB* (Gooderham et al., 2008), and overexpression of *pprB* resulted in an increased susceptibility to tobramycin under biofilm growth conditions (de Bentzmann et al., 2012). As the transcript levels of *psrA*, *pprB* and *pprA* were elevated in the absence of ProQ*_Pae_* (Figure 2A), we next asked whether ProQ impacts the sensitivity toward aminoglycosides in biofilms by determining the MBC of biofilm cells for tobramycin and gentamicin in PAO1 and PAO1Δ*proQ*. Biofilms were formed on 4 mm glass beads for 24 h in LB medium and then treated with different concentrations of tobramycin and gentamicin for 20 h. Survivor cells on beads were quantified by counting the CFUs. PAO1Δ*proQ* biofilms were more susceptible to tobramycin (Figure 4) and gentamicin (Supplementary Figure S6) than PAO1 biofilms. However, despite the elevated levels of *pprB* in PAO1∆*proQ* and at variance with de Bentzmann et al. (2012), we did not observe an increased susceptibility toward tobramycin of planktonically growing PAO1∆*proQ* (not shown). This might be explained by the overexpression of *pprB* from a P*_tac_* promoter (de Bentzmann et al., 2012), when compared to the rather moderately increased levels of *pprB* in PAO1∆*proQ* (Table 1).

### Increased susceptibility to sub-inhibitory concentration of GW-Q6 in the absence of ProQ*_Pae_*

**Figure 5:**
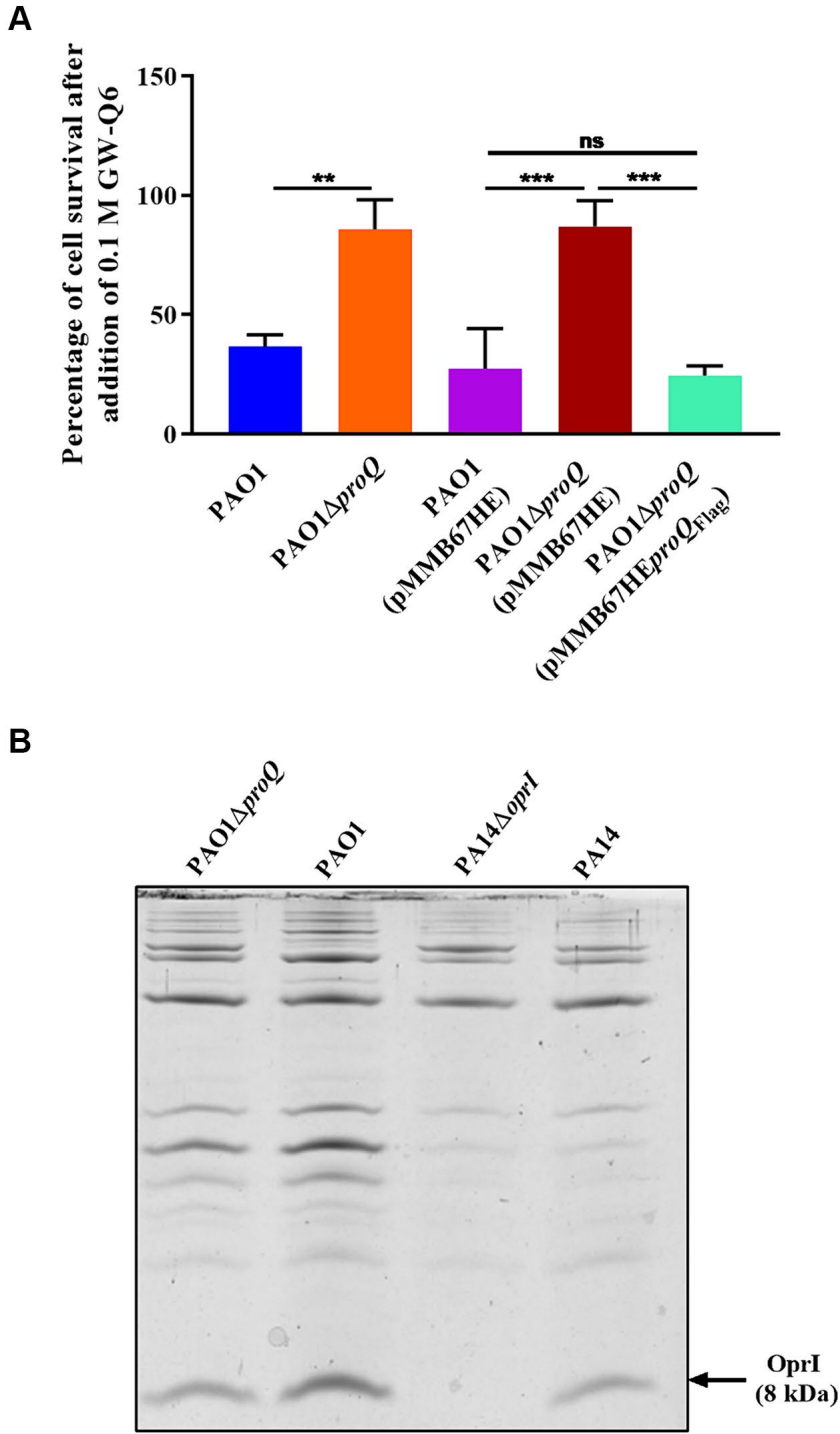
**(A)** Survival after exposure to the synthetic cationic AMP GW-Q6. PAO1 (blue), PAO1Δ*proQ* (orange), PAO1(pMMB67HE) (magenta), PAO1Δ*proQ*(pMMB67HE) (red) and PAO1Δ*proQ*(pMMB-*proQ*_Flag_) (cyan) were grown aerobically in LB medium to an OD_600_ of 2.0. Then, the cultures were diluted to approximately 10^4^ cells and treated with 0.1 μM of GW-Q6 peptide for 1.5 h. The percentage of cell survival was determined as described in Materials and Methods. The error bars represent standard deviations of two independent experiments. Statistical significance was determined using one-way ANOVA with the Tukey’s *post hoc* test. ns (non-significant), ^∗∗^*p* < 0.01 and ^∗∗∗^*p* < 0.001. **(B)** Determination of OprI protein levels. The strains PAO1, PAO1Δ*proQ*, PA14Δ*oprI* and PA14 were grown in LB medium. At an OD_600_ of 2.0, the cells were harvested, and the OM proteins were purified. The OM proteins were loaded on a 15.3% Tricine-SDS-polyacrylamide gel containing urea and stained with Coomassie Brilliant Blue. The arrow marks the position of the OprI protein.

The highly abundant OM lipoprotein OprI is targeted by naturally derived cationic AMPs such as SMAP-29, LL37 and human RNase7 in *Pae* (Lin et al., 2010). However, naturally occurring AMPs have low bioavailability and are prone to degradation (Moncla et al., 2011; Torcato et al., 2013). Therefore, synthetic AMPs were developed as a promising alternative strategy to combat multidrug-resistant pathogens (Chou et al., 2008; Lima et al., 2021). One of these newly designed cationic α-helical peptides, the synthetic AMP GW-Q6, has been shown to exert bactericidal activity in *Pae* by targeting OprI (Tseng et al., 2016). The transcript levels of *oprI* were 2.7-fold decreased in the absence of ProQ*_Pae_* (Table 1; Supplementary Table S3) suggesting that PAO1Δ*proQ* might exhibit increased resistance toward GW-Q6. To test this hypothesis, both strains were grown in LB medium to an OD_600_ of 2.0 and treated with a sub-inhibitory concentration of GW-Q6 for 1.5 h at 37°C (Figure 5A). The addition of the peptide reduced the survival of the wild-type strain by approximately 60%, while the lack of ProQ rendered the cells more resistant to the peptide, showing only a slight decrease in cell viability. Complementation of *proQ* through ectopic expression of the plasmid borne *proQ_Flag_* gene in strain PAO1Δ*proQ*(pMMB-*proQ*_Flag_) resulted again in increased sensitivity, whereas the presence of the empty vector pMMB67HE in PAO1 and PAO1Δ*proQ* showed the same susceptibility as the respective strains without plasmid (Figure 5A).

To confirm that the increased survival of PAO1Δ*proQ* strain was indeed due to a diminished amount of OprI protein, the OM proteins of PAO1 and PAO1Δ*proQ* were purified as described in Materials and Methods and separated on a Tris-Tricine-Urea-PAGE gel followed by Coomassie Brilliant Blue staining (Figure 5B). To unambiguously identify the 8 kDa OprI protein, the OM proteins of PA14 and of the PA14Δ*oprI* strain (grown under the same experimental conditions) were loaded as a positive and a negative control, respectively. The absence of ProQ*_Pae_* in strain PAO1Δ*proQ* resulted in an approximately 55% reduction of the OprI protein levels (Figure 5B).

## Discussion

The comparative transcriptome analysis clearly indicated that ProQ*_Pae_* acts – like its enterobacterial counterparts—as a regulator in *Pae* (Table 1; Supplementary Table S3). However, when compared with *E. coli* and *S. enterica*, the regulation of specific cellular functions by ProQ*_Pae_* seems to vary. In *E. coli* and *S. enterica*, ProQ is involved in osmoregulation, motility, and biofilm formation (Sheidy and Zielke, 2013; Kerr et al., 2014; Westermann et al., 2019). None of these processes seem to be affected by ProQ*_Pae_* (Supplementary Figure S2). Furthermore, some functions related to ProQ*_Nme_* (e.g., response to oxidative stress) were not affected by ProQ*_Pae_* (Supplementary Figure S2). Rather, ProQ*_Pae_* appears to control gene functions important for membrane integrity/permeability and antibiotic resistance in *Pae*.

We provided evidence that ProQ*_Pae_* affects antimicrobial susceptibility most likely through modulating the transcript levels of *psrA*, *pprA*, *pprB*, and *oprI*. There is only little information on FinO/ProQ-family proteins being involved in regulation of resistance functions. In most cases, the underlying mechanism seems to be related to the FinO/ProQ-family protein-mediated regulation of conjugation or replication of plasmids that contain antibiotic resistance genes (Dempsey, 1987; Gerovac et al., 2020; Yang et al., 2021). In *S. enterica*, ProQ*_Sen_* is involved in persister cell formation (Rizvanovic et al., 2022). ProQ*_Sen_* was shown to activate genes required for flagellum synthesis as well as genes of the pathogenicity island 2 (SPI-2), encoding a type III secretion system being important for intracellular survival. The enhanced expression of these genes causes an energetic burden, resulting in growth arrest of a subset of cells that are able to survive treatment with lethal concentrations of different antibiotics (Rizvanovic et al., 2022). We did not observe any difference in the swimming and swarming behavior of PAO1 and PAO1∆*proQ* (Supplementary Figure S2C), which are flagellum dependent (Henrichsen, 1972; Köhler et al., 2000). Thus, it seems rather unlikely that ProQ*_Pae_* affects persister cell formation by modulating flagellum biosynthesis (Supplementary Figure S2C).

PsrA was previously shown to be a positive regulator of type III secretion in a mucoid strain of *Pae* grown in complex medium (Shen et al., 2006). However, Gooderham et al. (2008) showed that PsrA is a negative regulator of type III secretion in the non-mucoid strain PAO1, and that a *psrA* mutant did not affect cytotoxicity toward epithelial cells, which is partially dependent on type III secretion. To our knowledge there is no evidence that PsrA is involved in persister cell formation in strain PAO1. It therefore remains elusive whether ProQ*_Pae_* can affect persister cell formation through modulation of the *psrA* transcript levels.

PsrA can act as a positive regulator of *pprB.* As shown in Figure 4 and Supplementary Figure S6, PAO1∆*proQ* showed an increased susceptibility toward tobramycin and gentamicin in biofilms, which can be reconciled with the elevated transcript levels of *psrA*, *pprA* and *pprB* (Figure 2A). The activation of the PprA/PprB TCS by PsrA results in an increased membrane permeability, which in turn leads to an increased sensitivity to tobramycin that is prevalent during biofilm conditions (de Bentzmann et al., 2012).

The activation of PprA/PprB in the absence of ProQ*_Pae_* is also in agreement with the increased expression of *cupE1* and *bapA* (Table 1; Figure 2A), which are known to be under positive control of the TCS (Bernard et al., 2009; Giraud et al., 2011; de Bentzmann et al., 2012). As the *psrA* and *pprB* mRNAs interact with ProQ-Strep (Figure 3B), it is likely that ProQ*_Pae_* regulate *pprB* directly by binding to its mRNA and indirectly by modulating PsrA-mediated transcriptional regulation of *pprB*. Moreover, in the absence of ProQ*_Pae_* a reduced abundance of the *speD2* and *speE2* genes was observed (Table 1). These functions are involved in spermidine biosynthesis and might also contribute to tobramycin resistance by altering the membrane permeability (Johnson et al., 2012; Wilton et al., 2015). In any case, the reduced abundance of these transcripts in the absence of ProQ*_Pae_* would be in accord with the observation that a deletion of *speE2* resulted in an increased aminoglycoside susceptibility in the presence of extracellular DNA, which contributes to biofilm formation (Whitchurch et al., 2002; Wilton et al., 2015).

The small major OM protein OprI plays a critical role in maintaining the integrity of the OM and serves as receptor for cationic α-helical AMPs such as the synthetic peptide GW-Q6 (Mizuno and Kageyama, 1979; Lin et al., 2010; Tseng et al., 2016). Binding of GW-Q6 to OprI causes a depolarization of the membrane and increases the membrane permeability (Tseng et al., 2016). Here, we have shown that the absence of ProQ*_Pae_* resulted in reduced *oprI* transcript levels (Figure 2B), and consequently OprI protein (Figure 5B). These results are consistent with the finding that the PAO1∆*proQ* strain displays an increased resistance toward sub-inhibitory concentration of the cationic AMP GW-Q6 (Figure 5A).

The co-purification studies with Strep-tagged ProQ*_Pae_* indicate that the protein associates with the *psrA*, *pprB*, *cupE1* and *oprI* transcripts (Figure 3B). However, it remains to be shown how the protein affects their transcript abundance. For some ProQ homologs, it has been suggested that the protein binds to the 3′-ends of mRNAs and stabilizes these transcripts (Holmqvist et al., 2018; Bauriedl et al., 2020; Gulliver et al., 2022; Bergman et al., 2024). Alternatively or in addition, ProQ*_Pae_* might be involved in sRNA-mediated regulation of these genes. This was shown for RaiZ-mediated regulation of *hupU* mRNA in *S. enterica*. Here, ProQ*_Sen_* stabilizes the sRNA RaiZ and facilitates duplex formation between RaiZ and *hupU*, which results in translational repression (Smirnov et al., 2017). In turn, the lack of translation is known to destabilize transcripts (Deana and Belasco, 2005; Kaberdin and Bläsi, 2006).

We did not observe any sRNA transcript to be affected by ProQ*_Pae_* (Supplementary Table S3). However, as only a limited number of sRNAs are annotated in the *Pae* genome database (Winsor et al., 2016), we cannot exclude that as yet unknown sRNAs are concerned or that the function but not the stability of the sRNAs are affected by ProQ*_Pae_*.

In summary, this study provided evidence that ProQ*_Pae_* can act as an RBP and regulator of antibiotic resistance determinants. However, whether ProQ*_Pae_* affects the susceptibility to antibiotics in a positive or negative manner seems to vary with the antibiotic class (e.g., AMPs or aminoglycosides). Hence, a better understanding of the underlying molecular mechanism(s) by which ProQ*_Pae_* regulates the respective mRNAs might offer novel strategies to counteract antibiotic resistance of *Pae*.

## Data availability statement

The datasets presented in this study can be found in online repositories. The names of the repository/repositories and accession number(s) can be found in the article/Supplementary material.

## Author contributions

AC: Writing – review & editing, Writing – original draft, Investigation, Formal analysis, Data curation, Conceptualization. AR: Writing – review & editing, Investigation, Formal analysis, Data curation. IM: Writing – review & editing, Conceptualization. UB: Writing – review & editing, Writing – original draft, Funding acquisition, Formal analysis, Conceptualization. ES: Writing – review & editing, Writing – original draft, Investigation, Funding acquisition, Formal analysis, Data curation, Conceptualization.

## Footnotes

^1^ http://www.csf.ac.at

^2^ http://www.bioinformatics.babraham.ac.uk/projects/fastqc/
