# Supplementary material for: The FinO/ProQ-like protein PA2582 impacts antimicrobial resistance in Pseudomonas aeruginosa
Source: Front Microbiol. 2024 Jun 26;15:1422742. doi: 10.3389/fmicb.2024.1422742 (PMC11247311; doi:10.3389/fmicb.2024.1422742)
Supplement: Supplementary file 1 [file Data_Sheet_1.pdf]

Supplementary Material

The FinO/ProQ-like protein PA2582 impacts antimicrobial resistance in *Pseudomonas aeruginosa*.

Anastasia Cianciulli Sesso[1,2], Armin Resch[1], Isabella Moll[1], Udo Bläsi[1]* and Elisabeth Sonnleitner[1]*

***Correspondence:**

Elisabeth Sonnleitner
elisabeth.sonnleitner@univie.ac.at

Udo Bläsi
udo.blaesi@univie.ac.at

SUPPLEMENTARY TEXT

1. Supplementary Materials and Methods

Biofilm Assay

For the static crystal violet assay (Merritt et al., 2005) diluted overnight cultures of PAO1 and PAO1Δ*proQ* were grown on 96-well microtiter plates for 24 h. Then, the contents of wells were removed, washed 3 times with water and air dried. 300 µl of 0.1% (w/v) crystal violet was added followed by incubation at room temperature for 10 min. The stain attached to the wells was solubilized using 95% ethanol. Biofilm formation was assessed by measuring the optical density of each sample at a wavelength of 595 nm.

Motility Assays

Swarming motility and Swimming motility were assayed as previously described (Déziel et al., 2001) on BM2-glucose plates supplemented with 0.5% casamino acids and 0.5% or 0.3% agar. Zone sizes were measured after overnight incubation at 37°C. Twitching motility was determined by sub-surface stab assays through LB plates containing 1% agar after overnight incubation at 37°C. Motility assays were carried out with two replicates and repeated at least three times.

Salt Stress Assay

The impact of sodium chloride (NaCl) on bacterial growth was assessed by growing *Pae* cultures on 96-well microtiter plates. Briefly, 200 µl LB medium containing either 0.17 M NaCl (no salt stress), 0.8 M (high salt) or 1 M NaCl (salt stress), were inoculated with freshly grown over-night cultures of PAO1 and PAO1Δ*proQ*, diluted to an initial OD_{600} of approximately 0.01. The growth was monitored by measuring the OD_{600} every hour by employing a Biotek Synergy H1 Hybrid Microplate Reader.

Oxidative Stress Assay

The impact of hydrogen peroxide (H_2O_2) on bacterial growth was assessed by a disc diffusion assay and carried out in duplicate. The cultures were grown in LB medium to an OD_{600} of 2.0 and spread

on the top of LB agar plates; disks containing 10 µl of 30% H_2O_2 were then placed onto the agar. The zone diameter of growth inhibition around each disk was measured after 24h incubation at 37°C.

Western-blot Analyses

Equal amounts of total proteins were separated on 12% SDS-polyacrylamide gels, and then electro-blotted onto a nitrocellulose membrane. The blots were treated with 5% dry milk in transfer buffer (25 mM Tris-base, 192 mM glycine, 0.1% (w/v) SDS, 20% (v/v) methanol). The blot was incubated with rabbit polyclonal antibodies directed against the Flag-tag epitope (DYKDDDDK; Antibodies Online GmbH). Anti-rabbit IgG coupled to horseradish peroxidase (Cell Signalling Technology) was used as a secondary antibody and the blot was developed using a chemiluminescent reagent (SuperSignal West Pico PLUS, Thermo Scientific). The signal was detected using the BioRad ChemiDoc™ MP Imaging system. Immunodetection of ribosomal protein S1 served as a loading control.

Determination of the Minimal Inhibitory Concentration (MIC) of Colistin by Evaluator Strips

Bacterial cultures of PAO1 and PAO1Δ*proQ* were grown in LB medium to an OD_{600} of 2.0. 100 µl (corresponding to approximately 3 x 10^8 CFU/ml) were plated on LB agar plates. Then, MIC Evaluator strips were applied with a concentration range of colistin between 0.016 and 256 µg/ml (Biomérieux).

Determination of the MIC of Tobramycin to Planktonically Growing Cells

The assay was performed in duplicate with strains PAO1 and PAO1Δ*proQ*, grown aerobically in LB medium to an OD_{600} of 2.0. Then, 0.5 ml of the culture was mixed with 1.5 ml of LB medium, containing serial dilutions of tobramycin (final concentrations from 0.25 to 64 µg/ml). The cultures were shaken at 37°C for additional 20 h. The minimal inhibitory concentrations corresponded to the lowest concentration of tobramycin that visibly impaired growth.

2 Supplementary Figures and Tables

2.1 Supplementary Tables

Supplementary Table S1. Strains and Plasmids used in this study.

Strains	Genotype/Relevant features	Reference
P. aeruginosa		
PAO1	Wild type	(Holloway et al., 1979)
PAO1Δ*proQ*		This study
PAO1Δ*hfq*		(Sonnleitner et al., 2017)
PAO1Δ*hfq*Δ*proQ*		This study
PAO1-$ProQ_{Flag}$	Encodes C-terminal Flag-tagged ProQ	This study
PAO1-$ProQ_{Strep}$	Encodes C-terminal Strep-tagged ProQ	This study
PA14	Wild type	(Liberati et al., 2006)
PAO1Δ*oprI*		(Tata et al., 2016)
E. coli		
S17-1	*pro, res⁻ hsdR17 (rK⁻ mK⁺) recA⁻ RP4-2-Tc::Mu-Km::Tn7*, Tp^R	(Simon et al., 1986)
**Plasmids**		
pMMB67HE	IncQ expression vector carrying an inducible P_{tac} promoter; Ap/Cb^R	(Fürste et al., 1986)
pMMB-*proQ*$_{Strep}$	pMMB67HE derivative encoding the C-terminal Strep-tagged ProQ protein. The corresponding gene is under transcriptional control of the P_{tac} promoter; Ap/Cb^R	This study
pMMB-*proQ*$_{Flag}$	pMMB67HE derivative encoding the C-terminal Flag-tagged ProQ protein. The corresponding gene is under transcriptional control of the P_{tac} promoter; Ap/Cb^R	This study
pEXG2	ColE1 suicide vector; mob *sacB* Gm^R	(Rietsch et al., 2005)
pEXG2-Δ*proQ*	pEXG2 with flanking regions to create an unmarked *proQ* (PA*2582*) deletion	This study
pEXG2-*proQ*$_{Strep}$	pEXG2 with flanking regions to create an in-frame fusion of *proQ* to a Strep-tag encoding sequence at the 3'-terminus	This study
pEXG2-*proQ*$_{Flag}$	pEXG2 with flanking regions to create an in-frame fusion of *proQ* to a Flag-tag encoding sequence at the 3'-terminus	This study

Supplementary Table S2. Oligonucleotides used in this study.

Designation	Sequence (5' → 3')[a]	Utilization
D181	TTTTTTT**CTGCAG**GGAGGAGGACCAGGCCAGG	PAO1Δ*proQ* and PAO1-ProQ$_{\mathrm{Flag}}$
E181	CCCTGCCCCGGCGA*TCA*CGTTCGCTTCACTAGTGCTG	PAO1Δ*proQ*
F181	TGATCGCCGGGGCAGGG	PAO1Δ*proQ*
G181	TTTTTTT**GAATTC**CAAGGCAAGATGAAACGGG	PAO1Δ*proQ*
I181	CCCTGCCCCGGCGA*TCACTTGTCGTCATCGTCTTTGTAGTC*GTTCGCCTCGGGGCTCGC	PAO1-ProQ$_{\mathrm{Flag}}$
H181	CCCTGCCCCGGCGA*TCATTTTTCGAACTGCGGGTGGCTCCAAGCGCT*GTTCGCCTCGGGGCTCGC	PAO1-ProQ$_{\mathrm{Strep}}$
P185	TTTTTTT**CTGCAG**GCACTAGTGAAGCGAACG	pMMB*proQ*$_{\mathrm{Strep}}$ and pMMB*proQ*$_{\mathrm{Flag}}$
Q185	TTTTTTT**GAATTC**GGCCCAGACGTCCGCG	pMMB*proQ*$_{\mathrm{Strep}}$ and pMMB*proQ*$_{\mathrm{Flag}}$
G196	TACCTGGAGGAGGTCTACGG	RT-qPCR: *psrA* (Forward)
H196	CAGCTTCGGCGCAGCCTCGTT	RT-qPCR: *psrA* (Reverse)
S194	CGGCAATATCCAGATCCAGT	RT-qPCR: *cupE1* (Forward)
T194	GAGATGTCCACCGGTGTGTT	RT-qPCR: *cupE1* (Reverse)
U194	TACGAGGTACACGGCAACAG	RT-qPCR: *pprB* (Forward)
V194	CCCAGCTCGTAGGCTATCTG	RT-qPCR: *pprB* (Reverse)
A195	GCGAATTGTGCGAGGTACA	RT-qPCR: *pprA* (Forward)
B195	TGCTTCACCTCGTGGGCG	RT-qPCR: *pprA* (Reverse)
Q117	AAGGCCCTGAAGAAGCACGG	RT-qPCR: *rpoD* (Forward)
R117	GATCGGCATGAACAGCTCGG	RT-qPCR: *rpoD* (Reverse)
I84	CAGCAATCTCCCCAGCCGGGG	Northern-blot: probe complementary to *oprI* mRNA
I26	CCCCACACTACCATCGGCGATGCGTCG	Northern-blot: probe complementary to 5S rRNA

a. Restriction sites are given in bold; Flag-tag and Strep-tag sequences are denoted in italics; Complementary sequences to F181 are underlined; Stop codons are highlighted in blue.

Supplementary Table S3. RNA_{Seq} based differential gene expression analysis of PAO1Δ*proQ versus* PAO1 grown in LB medium to an OD_{600} of 2.0. Genes with a fold-change equal or greater then ± 2 and padj ≥ of 0.05 are represented in bold. (Separate Excel file)

Supplementary Table S4. Co-localization of the $ProQ_{Pae}$ protein with potential RNA targets revealed by Grad-seq analysis performed by Gerovac et al. (2021) that were also identified by the RNA_{Seq} approach (**Supplementary Table S3**).

PA-number	Gene/Protein name	**1**	**2**	**3**	**4**	**5**	**6**	**7**	**8**	**9**	**10**	**11**	**12**	**13**	**14**	**15**	**16**	**17**	**18**	**19**	**20**	**P**
PA2582	ProQ	0,06	0,28	0,88	1,00	0,98	0,95	0,73	0,66	0,79	0,78	0,85	0,49	0,41	0,28	0,15	0,19	0,13	0,14	0,22	0,16	0,35
PA1874	*bapA*	0,22	0,65	0,97	0,78	1	0,61	0,89	0,61	0,19	0,16	0,2	0,13	0,17	0,02	0,13	0,09	0,02	0,19	0,06	0,05	0,31
PA2853	*oprI*	0,07	0,4	1	0,71	0,41	0,22	0,19	0,14	0,04	0,03	0,02	0,01	0,01	0,01	0,01	0,01	0,01	0,02	0,02	0,02	0,03
PA3006	*psrA*	0,16	0,37	0,83	0,88	1	0,67	0,87	0,88	0,34	0,31	0,25	0,17	0,11	0,08	0,07	0,07	0,07	0,08	0,1	0,07	0,25
PA3584	*glpD*	0,19	0,52	1	0,93	0,88	0,61	0,76	0,78	0,28	0,24	0,17	0,13	0,09	0,07	0,07	0,07	0,06	0,08	0,1	0,09	0,26
PA4293	*pprA*	0,3	0,47	0,43	1	0,81	0,4	0,55	0,61	0,25	0,18	0,07	0,12	0,04	0,09	0,12	0	0	0,03	0,18	0,05	0,3
PA4294	PA*4294*	0,11	0,39	1	0,5	0,17	0,35	0,63	0,47	0,13	0,17	0,04	0,02	0,06	0	0,14	0,04	0,07	0,03	0,03	0,06	0,03
PA4296	*pprB*	0,1	0,44	0,75	1	0,7	0,55	0,72	0,71	0,38	0,25	0,21	0,19	0,04	0,13	0,05	0,04	0,1	0,03	0,06	0,13	0,29
PA4596	*esrC*	0,07	0,27	0,65	1	0,66	0,52	0,55	0,73	0,24	0,25	0,15	0,14	0,06	0,05	0,01	0,05	0,04	0,05	0,05	0,04	0,13
PA4648	*cupE1*	0,05	0,29	0,39	0,92	0,52	0,66	1	0,66	0,43	0,31	0,23	0,15	0,05	0,05	0,05	0,09	0,08	0,13	0,1	0,08	0,28
PA4773	*speD2*	0,2	0,45	0,97	1	0,88	0,53	0,72	0,71	0,22	0,19	0,16	0,12	0,1	0,08	0,07	0,07	0,08	0,1	0,12	0,11	0,46
PA4774	*speD1*	0,17	0,39	0,8	0,85	0,93	0,61	0,87	1	0,4	0,39	0,31	0,25	0,18	0,14	0,14	0,14	0,15	0,2	0,23	0,21	0,83

The relative abundance is ranging from 0 to 1 and is shaded according to Gerovac et al. (2021).

2.2 Supplementary Figures

A

α1 α2

MGFEQLAELRDRLRAQAAQAKPAQTKSSAGRAKKREAVEPGVEAIWRLQRHFPLAFPKSP 60

α3 α4 α5

AAKVPLKQGILQDAQQHLELLGITAEQLKQAIATWCQGSRYWSCMVEDAPRLDLQGQVAG 120

α6

KVTAEQAVYARRQASRRQREQMREKRAKRAQAGGEAPAATEAPTPEAPATEASPEAN 177

B

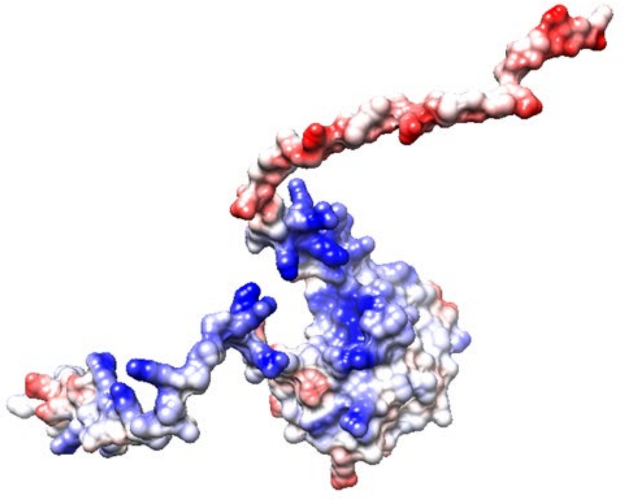

Supplementary Figure S1. (A) Protein sequence of ProQ*Pae*. Conserved residues important for RNA binding in *E. coli* (Stein et al., 2023) are shown in blue. Moieties that are predicted to form α-helices are indicated by red bars. (**B**) The electrostatic potential of ProQ*Pae* was calculated according to Coulomb's law using the UCSF Chimera software (Pettersen et al., 2004). The color depicts the electrostatic potential and ranges from -10 (red), over 0 (white) to +10 (blue) kcal/(mol*e).

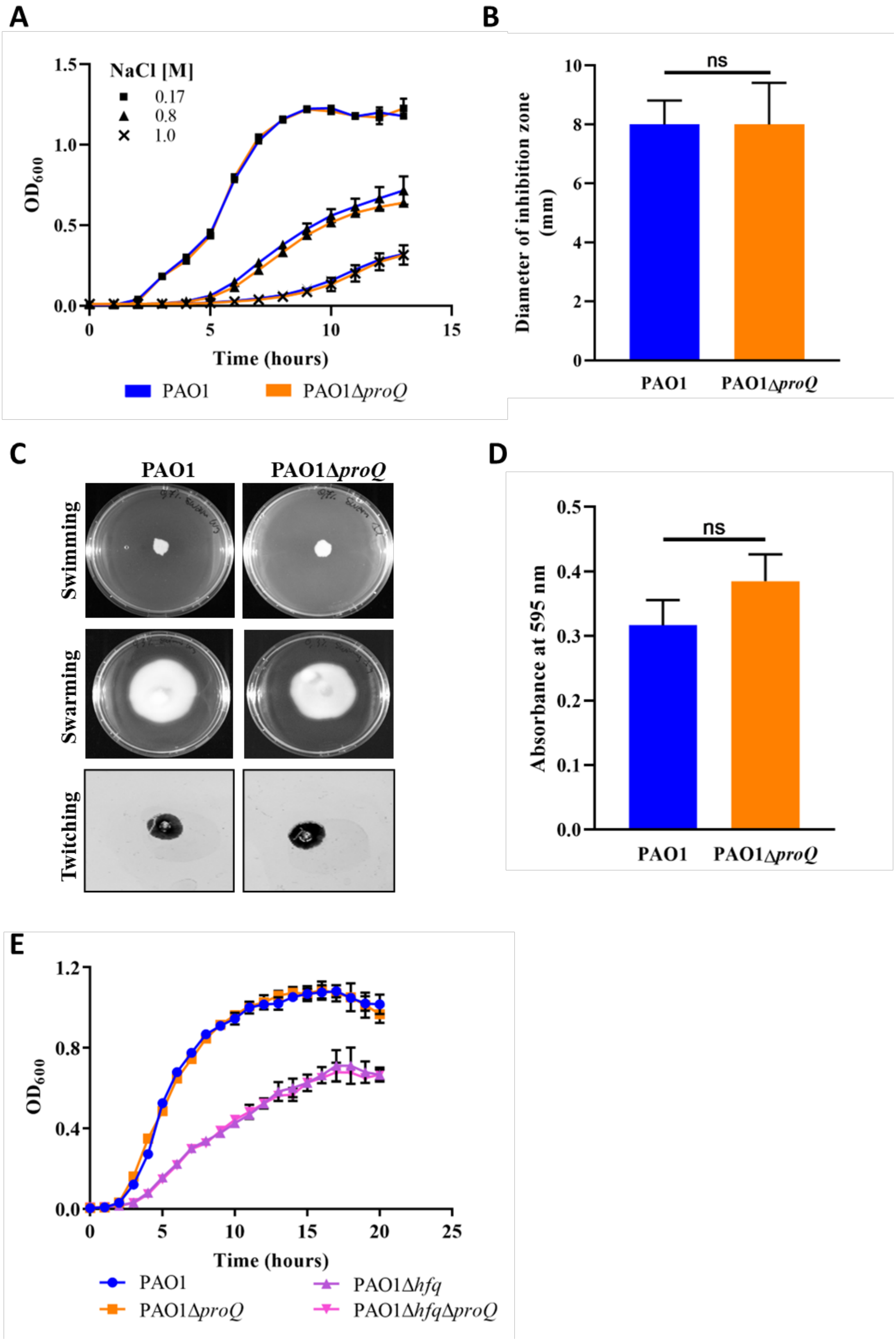
A
NaCl [M]
0.17
0.8
1.0
OD600
Time (hours)
PAO1
PAO1ΔproQ
B
ns
Diameter of inhibition zone (mm)
PAO1
PAO1ΔproQ
C
PAO1
PAO1ΔproQ
Swimming
Swarming
Twitching
D
ns
Absorbance at 595 nm
PAO1
PAO1ΔproQ
E
OD600
Time (hours)
PAO1
PAO1ΔproQ
PAO1Δhfq
PAO1ΔhfqΔproQ

Supplementary Figure S2. Phenotypic analyses of the PAO1Δ*proQ* strain. (**A**) Salt stress: PAO1 and PAO1Δ*proQ* were grown on a 96-well microtiter plate in LB medium containing either 0.17 M NaCl (no salt stress), 0.8 M (high salt) or 1 M NaCl (salt stress). (**B**) Oxidative stress: The diameter of the inhibition zones for H_2O_2 was determined by a disc diffusion assay. PAO1 and PAO1Δ*proQ* were grown in LB medium to an OD_{600} of 2.0 and then 100 µl of the cells were plated on LB plates followed by the application of discs containing 10 µl of a 30% H_2O_2 dilution. The diameter of the inhibition zone was determined after 24 h of growth at 37°C. (**C**) PAO1 and PAO1Δ*proQ* were grown over-night in LB medium and spotted onto BM2-glucose plates supplemented with 0.5% casamino acids and 0.5% (swarming) or 0.3% (swimming) agar. Twitching motility was assessed by spotting the strains on LB plates containing 1% agar. All the plates were incubated at 37°C for 24 h. (**D**) PAO1 and PAO1Δ*proQ* biofilms were grown under static conditions in LB medium for 24 h. The biofilms were stained with crystal violet, which was then eluted with ethanol, and the absorbance at 595 nm was measured. (**E**) PAO1, PAO1Δ*proQ*, PAO1Δ*hfq* and PAO1Δ*hfq*Δ*proQ* were grown in LB medium in a 96-well microtiter plates, and the OD_{600} was monitored continuously for 20 h. Error bars indicate standard deviations obtained from two biological replicates. In panels **(B)** and (**D)**, the significance was evaluated using a two-tailed Student's *t*-test and indicated as follows: ns (non-significant).

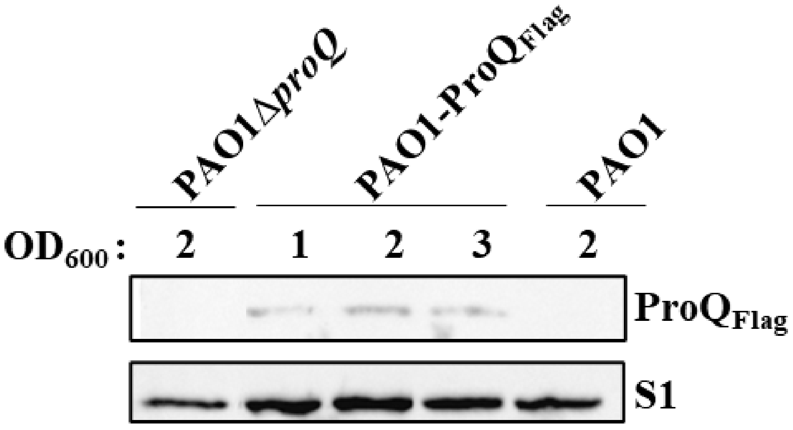

Supplementary Figure S3. Determination of ProQ*Pae* protein levels during growth in LB medium. The strains PAO1-ProQ$_{Flag}$, PAO1 and PAO1Δ*proQ* were grown in LB medium. PAO1-ProQ$_{Flag}$ cells were harvested at an OD_{600} of 1.0, 2.0 and 3.0, and samples of PAO1 and PAO1Δ*proQ* (negative controls) were taken at an OD_{600} of 2.0. The Flag-tagged ProQ*Pae* protein was identified by Western-blot analysis using anti-Flag antibodies. Immunodetection of ribosomal protein S1 served as a loading control**.**

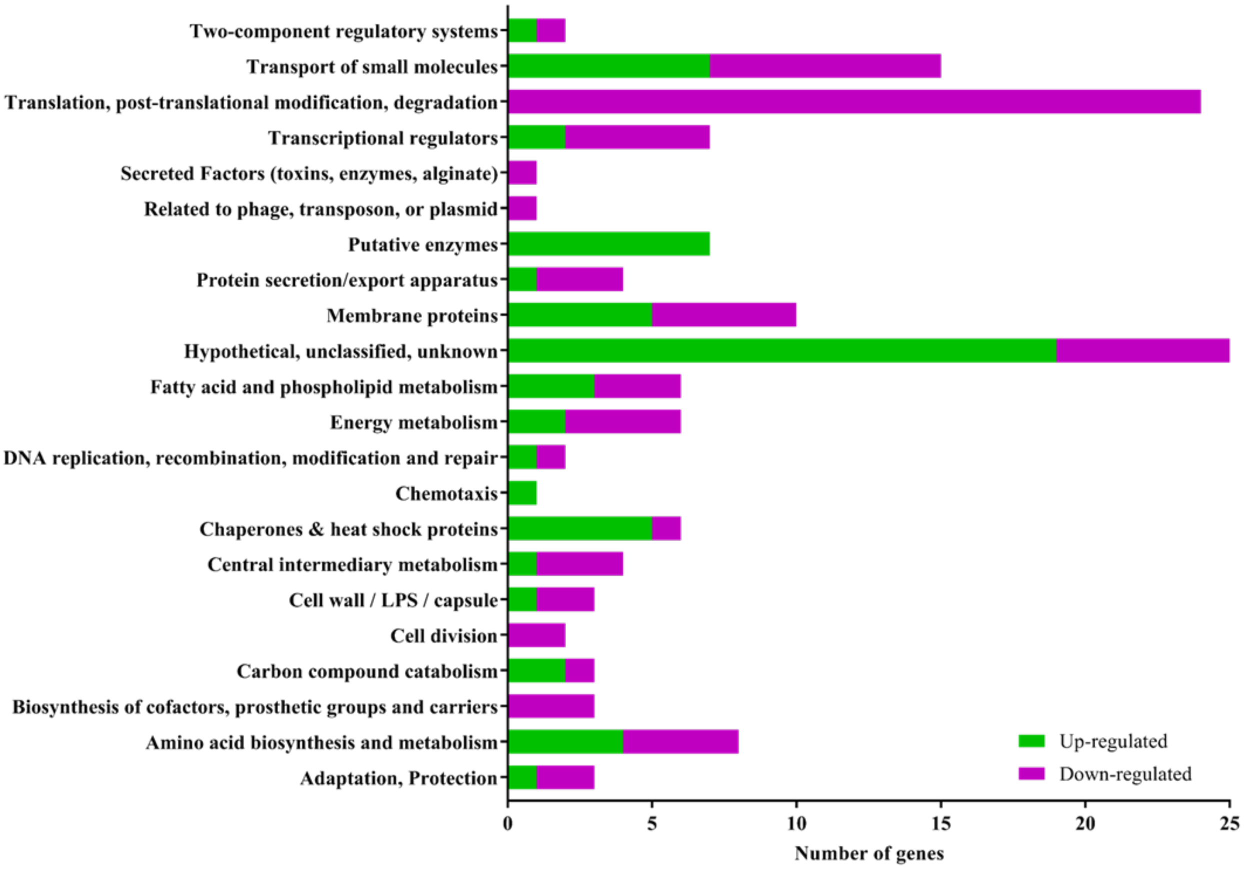

Supplementary Figure S4. PseudoCAP functional class distribution of annotated transcripts with altered abundance in PAO1Δ*proQ* when compared with PAO1. Green and violet bars indicate up- and down-regulated genes, respectively.

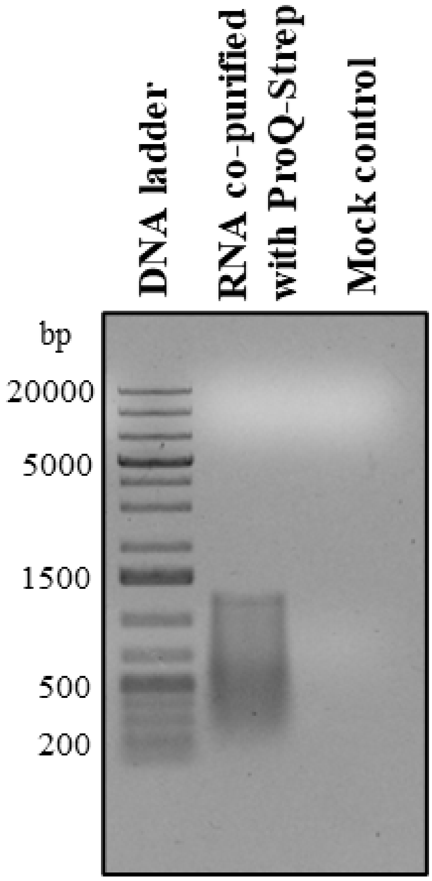

Supplementary Figure S5. Ethidium bromide-stained gel showing RNA co-purifying with Strep-tagged ProQ$_{Pae}$. PAO1Δ*proQ*(pMMB-*proQ*$_{\text{Strep}}$) and PAO1Δ*proQ*(pMMB67HE) (mock control) were grown in LB medium to an OD_{600} of 2.0. The RNA was isolated by phenol-chloroform extraction after purification of ProQ$_{Pae}$-Strep by affinity chromatography using the Strep-Tactin® resin. The corresponding eluates of the mock control were obtained under the same conditions. The RNA was separated on a 1% agarose gel and visualized with ethidium bromide. A DNA ladder is shown on the left.

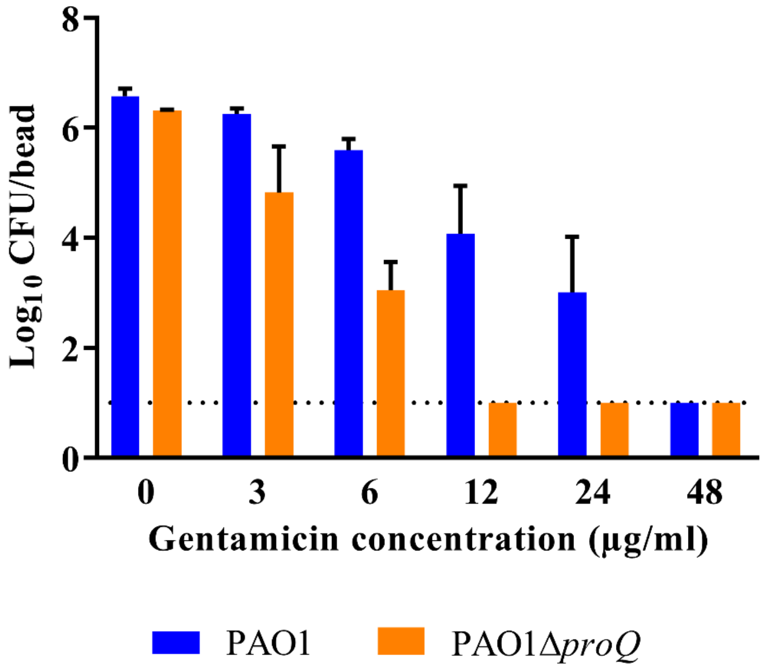

Supplementary Figure S6. Determination of the minimal bactericidal concentration (MBC) of gentamicin in biofilms. Biofilms of PAO1 and PAO1Δ*proQ* were grown at 37°C on 4 mm glass beads submerged in LB medium as described in **Materials and Methods**. Biofilm formation is displayed as the logarithm of the CFU per glass bead. Whenever the CFU count was zero, the value "1" (dashed line) was assigned. The error bars represent standard deviations from two biological replicates.

3. Supplementary References

Déziel, E., Comeau, Y., and Villemur, R. (2001). Initiation of biofilm formation by *Pseudomonas aeruginosa* 57RP correlates with emergence of hyperpiliated and highly adherent phenotypic variants deficient in swimming, swarming, and twitching motilities. *J. Bacteriol.* 183, 1195–1204. doi:10.1128/JB.183.4.1195-1204.2001.

Fürste, J.P., Pansegrau, W., Frank, R., Blocker, H., Scholz, P., Bagdasarian, M., et al. (1986). Molecular cloning of the plasmid RP4 primase region in a multi-host-range *tac*P expression vector. *Gene* 48**,** 119-131. doi: 10.1016/0378-1119(86)90358-6.

Holloway, B.W., Krishnapillai, V., and Morgan, A.F. (1979). Chromosomal genetics of *Pseudomonas*. *Microbiol. Rev.* 43**,** 73-102.

Liberati, N.T., Urbach, J.M., Miyata, S., Lee, D.G., Drenkard, E., Wu, G., et al. (2006). An ordered, nonredundant library of *Pseudomonas aeruginosa* strain PA14 transposon insertion mutants. *Proc. Natl. Acad. Sci. U. S. A.* 103, 2833-2838. doi: 10.1073/pnas.0511100103.

Merritt, J. H., Kadouri, D. E., and O'Toole, G. A. (2005). Growing and Analyzing Static Biofilms. *Curr. Protoc. Microbiol.* Chapter 1, Unit 1B.1. doi: 10.1002/9780471729259.MC01B01S00.

Pettersen, E. F., Goddard, T. D., Huang, C. C., Couch, G. S., Greenblatt, D. M., Meng, E. C. et al. (2004). UCSF Chimera - a visualization system for exploratory research and analysis. *J. Comput. Chem*., 25, 1605–1612. doi: 10.1002/jcc.20084.

Rietsch, A., Vallet-Gely, I., Dove, S.L., and Mekalanos, J.J. (2005). ExsE, a secreted regulator of type III secretion genes in *Pseudomonas aeruginosa*. *Proc. Natl. Acad. Sci. U. S. A.* 102**,** 8006-8011. doi: 10.1073/pnas.0503005102.

Simon, R., O'Connell, M., Labes, M., and Puhler, A. (1986). Plasmid vectors for the genetic analysis and manipulation of rhizobia and other gram-negative bacteria. *Methods Enzymol* 118**,** 640-659. doi: 10.1016/0076-6879(86)18106-7.

Sonnleitner, E., Prindl, K., and Bläsi, U. (2017). The *Pseudomonas aeruginosa* CrcZ RNA interferes with Hfq-mediated riboregulation. *PLoS One* 12**,** e0180887. doi: 10.1371/journal.pone.0180887.

Tata, M., Wolfinger, M.T., Amman, F., Roschanski, N., Dotsch, A., Sonnleitner, E., et al. (2016). RNASeq Based Transcriptional Profiling of *Pseudomonas aeruginosa* PA14 after Short- and Long-Term Anoxic Cultivation in Synthetic Cystic Fibrosis Sputum Medium. *PLoS One* 11**,** e0147811. doi: 10.1371/journal.pone.0147811.
